# Associations of Fatty Liver Disease with Hypertension, Diabetes, and Dyslipidemia: Comparison between Alcoholic and Nonalcoholic Steatohepatitis

**DOI:** 10.1155/2017/9127847

**Published:** 2017-08-22

**Authors:** Nobuyuki Toshikuni, Mutsumi Tsuchishima, Atsushi Fukumura, Tomiyasu Arisawa, Mikihiro Tsutsumi

**Affiliations:** ^1^Department of Hepatology, Kanazawa Medical University, 1-1 Daigaku, Uchinada-machi, Ishikawa 920-0293, Japan; ^2^Department of Gastroenterology, Kanazawa Medical University, 1-1 Daigaku, Uchinada-machi, Ishikawa 920-0293, Japan

## Abstract

Alcoholic steatohepatitis (ASH) and nonalcoholic steatohepatitis (NASH) are representative types of fatty liver disease (FLD) and have similar histologic features. In this study, we aimed to compare the associations of the two FLD types with hypertension (HT), diabetes mellitus (DM), and dyslipidemia (DL). A nationwide survey investigating FLD status included 753 Japanese subjects (median age 55 years; male 440, female 313) with biopsy-proven ASH (*n* = 172) or NASH (*n* = 581). We performed a multiple logistic regression analysis to identify the factors associated with HT, DM, or DL. Older age and a higher body mass index were significant factors associated with HT. Older age, female sex, a higher body mass index, advanced liver fibrosis, and the NASH type of FLD (odds ratio 2.77; 95% confidence interval 1.78–4.31; *P* < 0.0001) were significant factors associated with DM. Finally, the NASH type of FLD (odds ratio 4.05; 95% confidence interval 2.63–6.24; *P* < 0.0001) was the only significant factor associated with DL. Thus, the associations of NASH with DM and DL were stronger than those of ASH with DM and DL. In the management of FLD subjects, controlling DM and DL is particularly important for NASH subjects.

## 1. Introduction

Alcoholic liver disease (ALD) and nonalcoholic fatty liver disease (NAFLD) are two major types of fatty liver disease (FLD) [1]. Many studies have revealed the similar histologic findings of the two FLD types: both encompass simple steatosis (alcoholic fatty liver [AFL] and nonalcoholic fatty liver [NAFL]), steatohepatitis (alcoholic steatohepatitis [ASH] and nonalcoholic steatohepatitis [NASH]), progressive fibrosis, cirrhosis, and hepatocellular carcinoma [2, 3]. Moreover, epidemiological studies have suggested the close association of the two types of FLD with cardiovascular disease (CVD) [4–7]. Hypertension (HT), diabetes mellitus (DM), and dyslipidemia (DL) are well-known risk factors underlying CVD [8]. Both ALD and NAFLD are frequently associated with HT, DM, and DL: NAFLD is considered the hepatic manifestation of the metabolic syndrome, which includes two or more of the following factors: high blood pressure, high blood glucose, and DL [9, 10]. On the other hand, some studies have indicated the close associations of ALD with HT, DM, and DL [11–14]. However, few studies have compared the associations of the two FLD types with HT, DM, and DL. Analysis results from such studies will be helpful for preventing CVD in FLD patients.

In a previous study based on a nationwide survey of FLD status, we reported some differences in the associations of AFL and NAFL with HT, DM, and DL: the association of AFL with HT was stronger than that of NAFL with HT and the association of NAFL with DL was stronger than that of AFL with DL [15]. Based on these results, we performed a second nationwide survey of ASH and NASH. The aim of the current study was to compare the associations of ASH and NASH with HT, DM, and DL.

## 2. Materials and Methods

### 2.1. Subjects

For the second nationwide survey, we send a questionnaire to 101 hospitals where gastroenterologists affiliated with the Japanese Society of Gastroenterology worked and had participated in the first nationwide survey. Using the answers to the questionnaire, we collected data on subjects with biopsy-proven ASH or NASH (including progressive fibrosis and cirrhosis) who visited the hospitals between 2009 and 2011. The questionnaire collected information on patients' age; sex; body mass index (BMI); laboratory test values; drinking history; the presence or absence of HT, DM, and DL; and histopathological findings of the liver. All the patients gave informed consent at the time of liver biopsy. This study was conducted in accordance with the guidelines of the Declaration of Helsinki.

### 2.2. Definitions of ASH and NASH

The gastroenterologists who answered the questionnaire diagnosed ALD based on the criteria defined by the Alcohol and Liver Research Group of the Ministry of Education (alcohol consumption ≥ 60 g/day for >5 years for males and ≥40 g/day for >5 years for females) [16] and diagnosed NAFLD according to the criteria proposed by the Asia-Pacific Working Party (APWP) for NAFLD (≤20 g/day as the upper limit of alcohol consumption) [17]. Each gastroenterologist made the decision regarding the need for a liver biopsy in clinical practice. The histologic stage of ASH was determined according to the internationally accepted criteria [2]. The stage of NASH was determined based on the criteria proposed by Brunt [18]. Experienced pathologists who worked at the 101 hospitals were blinded to the patient clinical data and diagnosed the severity of ASH and NASH among the enrolled patients.

### 2.3. Criteria for HT, DM, and DL

The criteria for HT defined by the Japanese Society of Hypertension guidelines for the management of hypertension include a systolic blood pressure ≥ 140 mmHg or a diastolic blood pressure ≥ 90 mmHg [19]. The criteria for DM defined by the Japan Diabetes Society include a fasting blood glucose ≥ 126 mg/dL or a random blood glucose ≥ 200 mg/dL [20]. The definition of DL was a serum low-density lipoprotein (LDL) cholesterol ≥ 140 mg/dL, a serum high-density lipoprotein (HDL) cholesterol < 40 mg/dL (for both sexes), or serum triglycerides ≥ 150 mg/dL, according to the criteria of the Japan Atherosclerosis Society [21]. In this study, we enrolled patients with HT, DM, or DL regardless of whether they received medication.

### 2.4. Statistical Analysis

Continuous variables were expressed as medians (25, 75 percentile). The Mann–Whitney *U* test and the chi-square test were used for continuous variables and categorical variables, respectively. We performed a multiple logistic regression analysis to identify the factors associated with HT, DM, or DL. The potential factors were age (per year), sex, BMI (<25 kg/m^2^ versus 25 to 30 kg/m^2^ or >30 kg/m^2^), liver fibrosis stage (F1/F2 versus F3/F4), and FLD type. Following a univariate analysis, we conducted a multivariate analysis using all the above potential factors. A *P* value of <0.05 was considered statistically significant. We performed all analyses using the STATA version 13.1 software program (STATA Corp, College Station, TX, USA).

## 3. Results

### 3.1. Baseline Characteristics of the Subjects

Sixty-five (64.4%) of the 101 hospitals answered the questionnaire, providing data for 760 FLD subjects (172 ASH and 588 NASH). We excluded 7 NASH subjects because of missing data related to the presence or absence of HT, DM, and DL. Consequently, we enrolled 753 subjects (172 ASH and 581 NASH; median age 55 years; 440 male and 313 female).  Table 1 lists the subjects' baseline characteristics. NASH subjects were younger and had a lower male/female sex ratio and a higher BMI than ASH subjects. The prevalence of HT was similar between the two groups, while the prevalences of DM and DL were greater in NASH subjects than in ASH subjects. Furthermore, comparative analysis of the differences in the prevalences of the combinations of HT, DM, and DL revealed that the prevalences of HT + DM, HT + DL, DM + DL, HT + DM + DL, and all combinations of at least 2 of the 3 diseases were greater in NASH subjects than in ASH subjects.

### 3.2. Comparison of the Associations of ASH and NASH with HT, DM, and DL

We examined whether the two types of FLD, ASH and NASH, were associated with HT, DM, or DL. Regarding HT, the univariate analysis revealed that older age, a higher BMI, and advanced liver fibrosis were significant. On the multivariate analysis, older age and a higher BMI were significant factors associated with HT, while the FLD type was not associated with HT (Table 2). The univariate analysis found that older age, female sex, a BMI of >30 kg/m^2^, advanced liver fibrosis, and the NASH type of FLD were significantly associated with DM. The multivariate analysis identified the same factors as the univariate analysis (Table 3). Finally, the univariate analysis revealed that female sex, a higher BMI, advanced liver fibrosis, and the NASH type of FLD were significantly associated with DL. On the multivariate analysis, the NASH type of FLD was the only significant factor associated with DL (Table 4).

## 4. Discussion

The present study demonstrates that there are some differences in the associations of ASH and NASH with HT, DM, and DL: the associations of NASH with DM and DL were stronger than those of ASH with DM and DL. To our knowledge, this is the first study to reveal such differences between ASH and NASH patients. In our previous study of patients with simple steatosis (AFL and NAFL), we found that the association of AFL with HT was stronger than that of NAFL with HT and the association of NAFL with DL was stronger than that of AFL with DL [15]. Our two studies suggest that the associations of FLD with HT, DM, and DL may depend on the FLD type and the histologic stage. Regarding FLD and HT, AFL was more strongly associated with HT than NAFL, while ASH and NASH were similarly associated with HT. Regarding FLD and DM, AFL and NAFL were similarly associated with DM, whereas NASH was more strongly associated with DM than ASH was. Regarding FLD and DL, NAFL and NASH were more strongly associated with DL than AFL and ASH were, respectively. Thus, with the progression of the FLD stage, NAFLD subjects seemed to have more CVD risk factors than ALD subjects did.

We believe that the findings of this study are applicable in clinical practice. Our results were obtained from analyses of clinical and histologic data, whereas there are clinical data-derived indices for the histologic stage of FLD. For example, the aspartate aminotransferase/platelet ratio index (APRI) [22], the fibrosis-4 (FIB-4) index [23], and the FibroTest [24] have been validated for assessing the liver fibrosis stage in both ALD and NAFLD subjects. By using our findings and these indices, clinicians can, to some degree, estimate the associations of FLD with HT, DM, and DL according to the FLD type.

Our results may be linked to some differences in outcomes between ALD and NAFLD subjects. Few studies have directly compared the outcomes of ALD and NAFLD subjects. A recent cohort study, however, found that survival rates for ALD subjects were relatively low compared with those for NAFLD subjects [25]. Most studies examining the survival outcomes of FLD subjects have shown that CVD was the leading cause of death for NAFLD subjects [6, 26], while liver-related disease was the leading cause of death for ALD subjects [27]. Our findings of differences in the associations of ASH and NASH with DM and DL might, at least in part, explain the difference in the incidence of death due to CVD between these subjects. A comprehensive management strategy for both FLD per se and extrahepatic complications is required to improve the outcomes of FLD subjects; however, our findings and the reported outcomes of FLD subjects may suggest a difference in management priorities between the two types of FLD.

The present study of ASH and NASH subjects identified the factors associated with HT, DM, and DL. Older age and a higher BMI were associated with HT, consistent with the results of previous studies [28, 29]. In contrast, the FLD type was not a significant factor.

Older age and a BMI of >30 kg/m^2^ were associated with DM, results consistent with those of systematic analyses [30]. Furthermore, female sex was identified as a factor associated with DM. Epidemiological studies have shown that the prevalence of DM is generally higher in males than in females. However, the association between a high BMI and DM is stronger in females than in males [30]. This sex difference was also observed in our ASH and NASH subjects, more than half of whom had a BMI of ≥25 kg/m^2^. The present study, which included subjects with biopsy-proven ASH or NASH, found that advanced liver fibrosis was another factor associated with DM. This result agrees with those from studies examining the association of advanced liver fibrosis with DM [31, 32]. Furthermore, the FLD type of NASH was associated with DM, in contrast to HT. A similar result was reported in a study comparing ALD and NASH subjects [33]. Accumulating evidence has suggested that NALFD and DM may interact with each other: the presence of NAFLD predicts the development of DM; conversely, the presence of DM induces the progression of NAFLD [34]. A possible reason for the differences in the associations between DM and ASH and NASH is that ASH subjects may have less insulin resistance, a main facilitator of the pathogenesis of DM, than NASH subjects [35, 36]. Unfortunately, we did not collect data on the serum insulin levels of the enrolled subjects to analyze this point.

Our current and previous studies on the status of FLD revealed that NAFLD, irrespective of its histologic stage, is more strongly associated with DL than ALD is. A recent review investigating risk factors for CVD in subjects with chronic liver disease with various etiologies showed the following serum lipid profiles for ALD and NAFLD: among most ALD patients, serum LDL cholesterol levels, HDL cholesterol levels, and triglyceride levels are unaltered, increased, and increased, respectively, and among most NAFLD patients, serum LDL cholesterol levels, HDL cholesterol levels, and triglyceride levels are increased, decreased, and increased, respectively [37]. However, these results were obtained from studies of ALD and NAFLD, respectively. Our two studies directly comparing ALD and NAFLD subjects revealed that higher serum LDL cholesterol levels, conventional risk factors underlying CVD [38], were more clearly observed in NAFLD subjects than in ALD subjects. From the viewpoint of serum lipid profile, NAFLD subjects seem to have a higher risk of CVD than ALD subjects.

The present study has some limitations. First, this was a cross-sectional study; the design of which cannot prove the causality of the associations. Second, the present study used only Japanese subjects, which may hamper the generalizability of the findings. Third, patient selection bias might have affected the results because each gastroenterologist made the decision regarding the need for a liver biopsy in clinical practice. Fourth, no data on smoking were included in this study, which might have influenced our results. Many studies have examined the causal relationship between smoking and HT, DM, or DL, and some have demonstrated a positive relationship between smoking and DM [39]. It is possible that the percentage of smokers among the ASH subjects might be higher than that among the NASH subjects because habitual drinking is closely linked to habitual smoking [40]. If this assumption is correct, we may have overestimated the true association of ASH with DM. Nevertheless, our results revealed that the association of NASH with DM was stronger than that of ASH with DM. Thus, even if smoking had been included as a variable, the analyses would have provided the same results.

In conclusion, the present study suggests that the associations of NASH with DM and DL are stronger than those of ASH with DM and DL. These findings imply that NASH patients have more CVD risk factors than ASH subjects do. To improve the outcomes of FLD patients, it is pivotal to control both the risk factors and FLD per se. Our results indicate that NASH patients in particular require such control to prevent CVD.

## Figures and Tables

**Table 1:** Baseline characteristics of the enrolled subjects.

	Whole cohort (*n* = 753)	ASH (*n* = 172)	NASH (*n* = 581)	*P* value^∗^
Age, years	55 (43, 64)	58 (48, 66)	54 (42, 64)	0.0028
Sex, male/female	440/313	145/27	295/286	<0.0001
BMI, kg/m^2^	26.5 (23.5, 29.9)	22.1 (19.7, 24.5)	27.7 (24.8, 30.6)	<0.0001
BMI, <25/≥25 kg/m^2^, <30/≥30 kg/m^2^	286/282/185	131/34/7	155/248/178	<0.0001
Systolic blood pressure, mmHg	127 (116, 139)	130 (118, 140)	126 (116, 138)	0.056
Diastolic blood pressure, mmHg	78 (70, 85)	78 (70, 86)	78 (70, 85)	0.693
AST, IU/L	54 (35, 88)	61 (35.5, 137)	53 (35, 79.5)	0.0014
ALT, IU/L	67 (41, 115)	44 (27.5, 82)	73 (47.5, 120)	<0.0001
GGT, IU/L	73.5 (43, 156)	232.5 (121, 517.5)	62 (38, 101)	<0.0001
Fasting blood glucose, mg/dL	110 (98, 133)	113 (95, 134)	109 (98, 133)	0.705
Hemoglobin A1c, %	6.1 (5.6, 6.9)	5.6 (5.0, 6.5)	6.2 (5.7, 7.1)	<0.0001
Total cholesterol, mg/dL	194 (167, 226)	170 (138, 224)	198 (175, 226)	<0.0001
LDL cholesterol, md/dL^†^	114 (90, 139)	95 (59, 124)	116 (94, 140)	0.0001
HDL cholesterol, mg/dL	48 (40, 58)	44 (31, 61)	49 (41, 58)	0.033
Triglycerides, mg/dL	134 (96, 206)	128 (89, 198)	135 (99, 207)	0.244
Stage of liver fibrosis, F1/F2/F3/F4	227/234/185/107	41/38/29/64	186/196/156/43	<0.0001
HT, *n* (%)	342 (45.4)	71 (41.3)	271 (46.6)	0.215
Without medication, *n* (%)	118 (34.5)	24 (33.8)	94 (34.7)	
With medication, *n* (%)	224 (65.5)	47 (66.2)	177 (65.3)	
DM, *n* (%)	349 (46.3)	47 (27.3)	302 (52.0)	<0.0001
Without medication, *n* (%)	185 (53.0)	35 (74.5)	150 (49.7)	
With medication, *n* (%)	164 (47.0)	12 (25.5)	152 (50.3)	
DL, *n* (%)	436 (57.9)	52 (30.2)	384 (66.1)	<0.0001
Without medication, *n* (%)	237 (54.4)	21 (40.4)	216 (56.2)	
With medication, *n* (%)	199 (45.6)	31 (59.6)	168 (43.8)	
HT + DM, *n* (%)	202 (26.8)	27 (15.7)	175 (30.1)	<0.0001
HT + DL, *n* (%)	218 (29.0)	24 (14.0)	194 (33.4)	<0.0001
DM + DL, *n* (%)	247 (32.8)	20 (11.6)	227 (39.1)	<0.0001
HT + DM + DL, *n* (%)	147 (19.5)	13 (7.6)	134 (23.1)	<0.0001
≥2 of the 3 diseases, *n* (%)	373 (49.5)	45 (26.2)	328 (56.5)	<0.0001

ASH: alcoholic steatohepatitis; NASH: nonalcoholic steatohepatitis; BMI: body mass index; AST: aspartate aminotransferase; ALT: alanine aminotransferase; GGT: *γ*-glutamyl transpeptidase; LDL: low-density lipoprotein; HDL: high-density lipoprotein; HT: hypertension; DM: diabetes mellitus; DL: dyslipidemia. ^∗^ASH versus NASH. Chi-square test for categorical variables, Mann–Whitney *U* test for continuous variables. ^†^The Friedewald equation was used. Data excluded 7 subjects with ASH and 12 with NASH whose serum triglyceride levels were ≥400 mg/dL.

**Table 2:** Associations of ASH and NASH with HT.

Variable	Univariate analysis			Multivariate analysis		
	OR	95% CI	*P* value	OR	95% CI	*P* value
Age, per year	1.05	1.03–1.06	<0.0001	1.05	1.04–1.07	<0.0001
Sex, male	0.84	0.63–1.13	0.244	1.35	0.96–1.91	0.085
BMI, ≥25 kg/m^2^, <30 kg/m^2^	1.58	1.13–2.20	0.007	1.74	1.19–2.55	0.004
BMI, ≥30 kg/m^2^	1.41	0.97–2.05	0.072	2.24	1.43–3.50	<0.0001
Liver fibrosis, F3/F4	1.41	1.05–1.90	0.021	1.10	0.79–1.52	0.584
FLD type, NASH	1.24	0.88–1.76	0.215	1.27	0.82–1.96	0.282

ASH: alcoholic steatohepatitis; NASH: nonalcoholic steatohepatitis; HT: hypertension; OR: odds ratio; CI: confidence interval; BMI: body mass index; FLD: fatty liver disease.

**Table 3:** Associations of ASH and NASH with DM.

Variable	Univariate analysis			Multivariate analysis		
	OR	95% CI	*P* value	OR	95% CI	*P* value
Age, per year	1.01	1.004–1.02	0.005	1.01	1.003–1.03	0.014
Sex, male	0.47	0.35–0.63	<0.0001	0.68	0.49–0.94	0.021
BMI, ≥25 kg/m^2^, <30 kg/m^2^	1.27	0.91–1.77	0.157	0.97	0.67–1.41	0.877
BMI, ≥30 kg/m^2^	2.02	1.39–2.95	<0.0001	1.56	1.01-2.41	0.043
Liver fibrosis, F3/F4	1.59	1.19–2.14	0.002	1.65	1.19–2.28	0.003
FLD type, NASH	2.88	1.98–4.18	<0.0001	2.77	1.78–4.31	<0.0001

ASH: alcoholic steatohepatitis; NASH: nonalcoholic steatohepatitis; DM: diabetes mellitus; OR: odds ratio; CI: confidence interval; BMI: body mass index; FLD: fatty liver disease.

**Table 4:** Associations of ASH and NASH with DL.

Variable	Univariate analysis			Multivariate analysis		
	OR	95% CI	*P* value	OR	95% CI	*P* value
Age, per year	0.99	0.98–1.001	0.115	1.00	0.99–1.01	0.728
Sex, male	0.68	0.51–0.92	0.012	0.92	0.65–1.29	0.613
BMI, ≥25 kg/m^2^, <30 kg/m^2^	1.70	1.22–2.38	0.002	1.05	0.72–1.53	0.798
BMI, ≥30 kg/m^2^	2.10	1.43–3.08	<0.0001	1.16	0.75–1.80	0.509
Liver fibrosis, F3/F4	0.71	0.53–0.95	0.023	0.86	0.62–1.20	0.378
FLD type, NASH	4.50	3.11–6.50	<0.0001	4.05	2.63–6.24	<0.0001

ASH: alcoholic steatohepatitis; NASH: nonalcoholic steatohepatitis; DL: dyslipidemia; OR: odds ratio; CI: confidence interval; BMI: body mass index; FLD: fatty liver disease.
